# Maternal Proteins That Are Phosphoregulated upon Egg Activation Include Crucial Factors for Oogenesis, Egg Activation and Embryogenesis in *Drosophila melanogaster*

**DOI:** 10.1534/g3.118.200578

**Published:** 2018-07-16

**Authors:** Zijing Zhang, Amber R. Krauchunas, Stephanie Huang, Mariana F. Wolfner

**Affiliations:** Department of Molecular Biology and Genetics, Cornell University, Ithaca, NY

**Keywords:** egg activation, oogenesis, embryogenesis, maternal protein, phosphorylation state, *Drosophila melanogaster*

## Abstract

Egg activation is essential for the successful transition from a mature oocyte to a developmentally competent egg. It consists of a series of events including the resumption and completion of meiosis, initiation of translation of some maternal mRNAs and destruction of others, and changes to the vitelline envelope. This major change of cell state is accompanied by large scale alteration in the oocyte’s phosphoproteome. We hypothesize that the cohort of proteins that are subject to phosphoregulation during egg activation are functionally important for processes before, during, or soon after this transition, potentially uniquely or as proteins carrying out essential cellular functions like those they do in other (somatic) cells. In this study, we used germline-specific RNAi to examine the function of 189 maternal proteins that are phosphoregulated during egg activation in *Drosophila melanogaster*. We identified 53 genes whose knockdown reduced or abolished egg production and caused a range of defects in ovarian morphology, as well as 51 genes whose knockdown led to significant impairment or abolishment of the egg hatchability. We observed different stages of developmental arrest in the embryos and various defects in spindle morphology and aberrant centrosome activities in the early arrested embryos. Our results, validated by the detection of multiple genes with previously-documented maternal effect phenotypes among the proteins we tested, revealed 15 genes with newly discovered roles in egg activation and early embryogenesis in Drosophila. Given that protein phosphoregulation is a conserved characteristic of this developmental transition, we suggest that the phosphoregulated proteins may provide a rich pool of candidates for the identification of important players in the egg-to-embryo transition.

At the end of oogenesis, mature oocytes arrest in meiosis with repressed transcription and a rich deposit of maternal mRNAs and proteins. For development to proceed, the oocyte needs to restart some quiescent cellular activities, and terminate other ongoing activities. This transition occurs through the process of egg activation, which consists of a series of cellular events including the resumption and completion of meiosis, modifications of the outer egg coverings, and dynamic regulation of maternal mRNAs and remodeling of the oocyte transcriptome and proteome (Horner & Wolfner 2008b, Krauchunas & Wolfner 2013, Laver *et al.* 2015, Machaty *et al.* 2017, Marcello & Singson 2010, Von Stetina & Orr-Weaver 2011). Egg activation is an intricate process through which the oocyte shifts gears from oogenesis to embryogenesis, from a differentiated gamete into a totipotent zygote that gives rise to all the cell types of the adult. Some of the regulatory molecules for this essential developmental transition have been identified genetically (*e.g.*, in Drosophila (Cui *et al.* 2016, Cui *et al.* 2008, Elfring *et al.* 1997, Freeman *et al.* 1986, Lee *et al.* 2003, Lim *et al.* 2016, Lin & Wolfner 1991, Renault *et al.* 2003, Tadros *et al.* 2007)), but the number is surprisingly small. It seems likely that many molecules essential for this transition cannot be identified via traditional maternal effect screens due to their essentiality for the organism’s survival.

Egg activation initiates with a rise of Ca^2+^ level in the cytoplasm of the oocyte. This calcium rise is triggered by fertilization in vertebrates and some invertebrates (Knott *et al.* 2005, Saunders *et al.* 2002, Singaravelu & Singson 2013, Steinhardt *et al.* 1977, Yoon *et al.* 2012). But in insects, the calcium rise is triggered by the passage of the oocyte through the reproductive tract, and is induced independent of fertilization (Horner & Wolfner 2008a, Kaneuchi *et al.* 2015, Sartain & Wolfner 2013, York-Andersen *et al.* 2015). Since no significant transcription occurs during oocyte maturation and egg activation (De Renzis *et al.* 2007, Newport & Kirschner 1982, Zalokar 1976), the dowry of maternal mRNAs and proteins deposited into the oocyte must include all the essential machinery to regulate the cellular events of egg activation in response to the calcium rise. Furthermore, a study in Drosophila found that meiosis can be completed even when translation is inhibited (Page & Orr-Weaver 1997). These findings suggest that the regulation of egg activation, and the early embryonic events that immediately follow, relies heavily on maternally-provided proteins. However, some of these proteins might need to be active at one stage in this transition (*e.g.*, oogenesis) and inactive at another (*e.g.*, early embryogenesis) or the reverse, and thus might be post-translationally modulated to accomplish this.

During egg activation, the oocyte proteome is dynamically regulated through degradation and post-translational modification (Guo *et al.* 2015, Krauchunas *et al.* 2012, Kronja *et al.* 2014a, Laver *et al.* 2015, Presler *et al.* 2017, Roux *et al.* 2006). Among the latter processes, changes in protein phosphorylation state are very prevalent during this transition. In *Drosophila melanogaster*, over 300 maternal proteins alter their phosphorylation state during egg activation (Krauchunas *et al.* 2012), including a few previously shown to be crucial for egg activation and the onset of syncytial divisions, such as Young arrest (YA) and Giant nuclei (GNU) (Lin & Wolfner 1991, Renault *et al.* 2003, Sackton *et al.* 2009, Yu *et al.* 1999). In sea urchins, changes at hundreds of phosphosites were reported to occur within 2 min and 5 min post fertilization (Guo *et al.* 2015, Roux *et al.* 2006), and in *Xenopus* ∼500 phosphosites show dynamic regulation following fertilization (Presler *et al.* 2017). Consistent with the remodeling of oocyte phosphoproteome seen during the egg-to-embryo transition, two highly-conserved calcium-dependent regulators that are essential for egg activation in several species, Ca^2+^/Calmodulin dependent kinase II (CaMKII) (*Xenopus* and mouse (Backs *et al.* 2010, Morin *et al.* 1994)) and calcineurin (*Xenopus* and Drosophila; (Horner *et al.* 2006, Mochida & Hunt 2007, Takeo *et al.* 2010, Takeo *et al.* 2006)), are a kinase and a phosphatase respectively (Hudmon & Schulman 2002, Rusnak & Mertz 2000). These findings suggest that phosphoregulation is a conserved, functionally important regulatory mechanism that modulates protein activities upon egg activation. Thus, the set of proteins that change in phosphorylation state at this time may be an excellent candidate pool for identification of factors involved in processes before, during and after egg activation, with phosphorylation being a rapid way to turn on/off their activities at the appropriate time. Such molecules could include ones specific to the female germline or the early embryo, or proteins needed commonly for cellular events later in development (but not yet tested for roles in the germline or egg activation).

Consistent with this hypothesis, 174 conserved proteins alter their phosphorylation states during the egg-to-embryo transition in both sea urchin and the Drosophila (Krauchunas *et al.* 2012, Guo *et al.* 2015), suggesting that a cohort of phosphoregulated proteins is deeply conserved, and may be functionally important for the transition from oocyte to embryo.

Here, we tested 189 proteins that are phosphoregulated upon egg activation in *Drosophila melanogaster* (Krauchunas *et al.* 2012) for their involvement in oocyte formation and/or the transition from oocyte to early embryo. The UAS-Gal4 system (Brand & Perrimon 1993) enabled us to dissect the function of our target genes by robust germline-specific RNAi knockdown of their expression (Ni *et al.* 2011), thus avoiding the lethality that could have arisen in a whole-organism mutational screen.

We identified 53 genes whose products are crucial for oogenesis. We also observed maternal effect phenotypes upon knockdown of 51 genes, 27 of which caused significant arrest in early embryogenesis, indicating vital roles of their products in the initiation of zygotic development. These genes encode factors known to be involved in various cellular processes in somatic cells, such as mitosis and metabolism. Twelve of these 27 genes have previously been reported to have maternal effects, thus validating our screen and indicating that the other 15 genes are likely new regulators of this process. We also show that many factors involved in mitosis and other processes in later somatic cells are also essential for the development of oocyte and the syncytial divisions in early embryos. Our study establishes the cohort of maternal proteins that are phosphoregulated during egg activation as a rich candidate pool for the identification of factors essential for female fertility.

## Materials and Methods

### Fly stocks

Fly stocks were maintained on standard yeast-glucose-agar media, at 23 ± 2°, under a 12-hr light-dark cycle.

### Germline-specific RNAi

A total of 207 transgenic shRNA lines (Ni *et al.* 2011, Perkins *et al.* 2015) carrying -WALIUM or VALIUM- vectors driving shRNA targeting 189 candidates were obtained from the Bloomington stock center, the NIG stock center, and the Vienna Drosophila Resource Center (stock numbers are listed in Table S1). 5-10 virgin females from the shRNA stock were mated to 5-10 males carrying either MTD-Gal4, which drives expression throughout oogenesis (Petrella *et al.* 2007), or *mata4*-GAL4-VP16, which drives expression after the germarium stages (Radford *et al.* 2012, Sugimura & Lilly 2006). These crosses generated females that are heterozygous for both shRNA and the driver construct (referred to here as “RNAi females”). Background-matched control females, heterozygous for the AttP2 (empty insertion site of the shRNA construct) and the appropriate driver construct were generated in parallel to serve as controls. RNAi females and control females were both raised and maintained at 27°.

### Fertility assays

Virgin RNAi females and control females were mated to Oregon R P2 (Allis *et al.* 1977) males in single pairs; mating was confirmed by observation. Males were removed after mating and females were allowed to lay eggs on standard fly media and were transferred to new culture vials every 24 hr for 4 days. The number of eggs laid in each vial was counted. The hatch rate was estimated as the proportion of eggs that developed into pupae. For each assay, RNAi females for 5-10 TRiP lines (5-10 RNAi females for each line) and 1 control group (10 control females) were included. Total 4-day egg production and overall embryo hatchability were calculated for each female. The 4-day egg production and embryo hatchability of the 5-10 RNAi females for each TRiP line were compared to those of the 10 control females from the same assay using Student’s T-test (Table S1). RNAi lines that generated fertility phenotypes were re-tested in 2-3 independent biological replicates, to ensure reproducibility of the results.

### Reverse-transcription PCR

Stage 14 oocytes were dissected in hypertonic Isolation Buffer, which prevents egg activation (Page & Orr-Weaver 1997). RNA was extracted using TRIzol (Thermo Fisher). RNA samples were treated with DNase (Promega) and reverse transcribed with SmartScribe Reverse Transcriptase Kits (CloneTech Laboratories, Inc). PCR was performed with cDNA samples using primers for the gene being tested for knockdown and primers for Rpl32 as control; primers are listed in Table S2. Band intensities of the amplicons from knockdown and control samples were quantified and compared using ImageJ, as described in Findlay *et al.* 2014 (Findlay *et al.* 2014). For each sample tested (knockdown, control) amplicon band intensity of the target gene was normalized to that of Rpl32. We focused our analysis on the knockdowns that gave 40% or less of control expression of the targeted gene (Sopko *et al.* 2014).

### Bioinformatics

Peptide sequences were scanned for consensus phosphorylation site using Scansite 2.0 Motif Scan (Obenauer *et al.* 2003). The result included consensus phosphorylation sites identified on all the isoforms of each protein.

### Immunofluorescence

2-4hr old and 0.5-1.5hr old fertilized embryos were collected on grape juice agar plates, de-chorionated with 50% bleach, fixed using heptane/methanol and stained as described in (Horner *et al.* 2006). Ovaries from 3-5-day old females were dissected in Isolation Buffer (Page & Orr-Weaver 1997) and fixed using Fixation Buffer (Radford & McKim 2016), incubated with 5µg/ml RNaseA overnight (Horner *et al.* 2006), and stained with propidium iodide. Mouse anti-αtubulin (Sigma, St. Louis, MO, catalog #T5168) was used at a dilution of 1:400. Mouse anti-γtubulin (Boster Biological Technologies, Pleasanton, CA, catalog #MA1114) was reconstituted in 1ml of PBS and used at a dilution of 1:100. Mouse anti-DROP-1, which stains sperm tails (Karr 1991), (kindly provided by T. Karr at Kyoto University) was used at 1:800 as described in Krauchunas *et al.* (2012). Alexa Fluor 488 conjugated anti-mouse (Thermo Fisher) was used at 1:200. Propidium iodide was used at 10 µg/ml. Samples were examined and images were generated using a Leica TCS SP2 confocal microscope at the Cornell Imaging Core.

### Immunoblotting

80-100 oocytes or activated but unfertilized eggs (collected from females mated to the spermless sons of *tudor* females; Boswell & Mahowald 1985) were homogenized in 10 µl of Protein Extraction Buffer (10 mM Tris, pH 7.5; 20 mM NaF, 2 mM EGTA, 10 mM DTT, 400 nM okadaic acid, and 2% SDS), and the lysate was mixed with 10 µl of SDS loading buffer. Proteins were separated by electrophoresis in 8% polyacrylamide SDS gels. Primary guinea pig anti-Smg antibody (Semotok *et al.* 2005) (kindly provided by H. Lipshitz at University of Toronto) was used at 1:10000. Mouse anti-tubulin was used at dilution of 1:10000 (Sigma, St. Louis, MO, catalog #T5168). Secondary HRP conjugated anti-guinea pig was used at 1:1000 (Jackson Laboratories). Anti-mouse secondary antibody was used at 1:2000 (Jackson Laboratories).

### Data availability

Strains and plasmids are available upon request. The authors affirm that all data necessary for confirming the conclusions of the article are present within the article, figures, and tables. Supplemental material is available at Figshare: https://doi.org/10.25387/g3.6820784.

## Results

### Germline-specific depletion of phosphoregulated maternal proteins reveals factors crucial for different aspects of female fertility

To test whether the subset of proteins that are phosphoregulated during egg activation are important for female fertility in *Drosophila melanogaster*, we obtained all available TRiP RNAi lines that targeted the phosphoregulated maternal proteins identified by Krauchunas *et al.* (2012).

A total of 207 TRiP lines targeting 189 genes were obtained. Utilizing the proteomic data published in a recent study of posttranscriptional changes during Drosophila egg activation (Kronja *et al.* 2014a), we examined the abundance changes of the proteins we studied. Of the 126 proteins with proteomic data available, the abundance of 112 did not change significantly during the transition from oocyte to embryo (Table S1). This supports our view that phosphoregulation is the mechanism that modulates these proteins at this transition.

**Figure 1:**
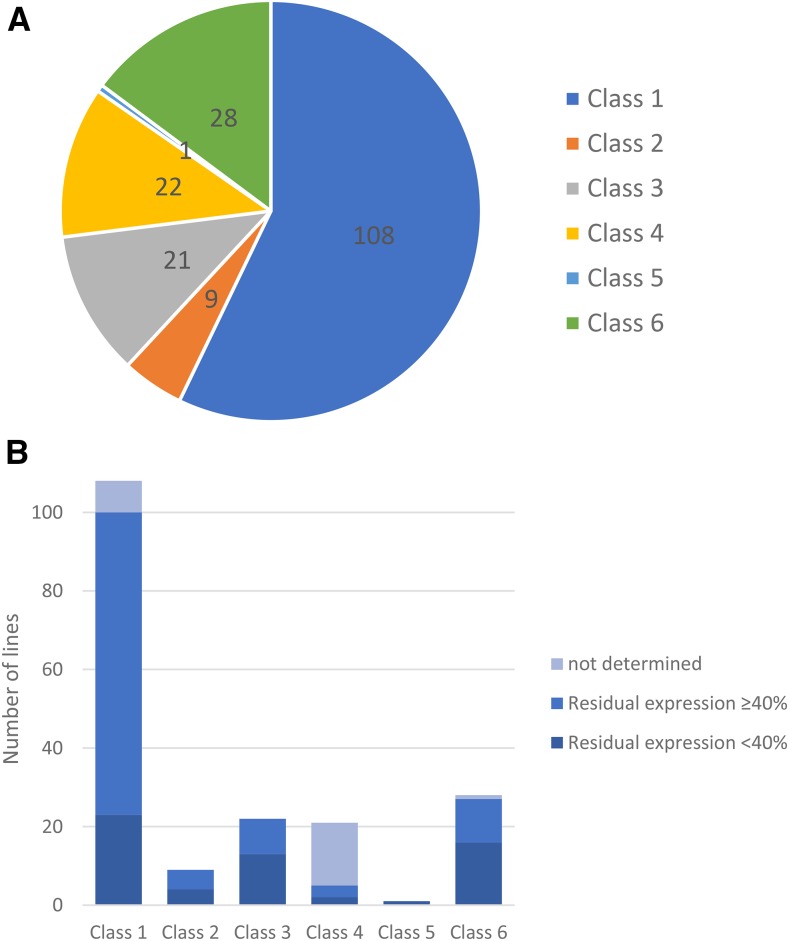
Germline-specific RNAi knockdown of maternal proteins that are phosphoregulated upon egg activation exhibited different impacts on female fertility. (A) The frequency of fertility phenotypes observed in RNAi females (MTD-Gal4/UAS-shRNA or matαrub-Gal4/UAS-shRNA) crossed to ORP2 males. A total of 189 genes were screened. (B) Knockdown efficiency of the target gene in different phenotype classes. The number of genes in each phenotypic class with residual expression level equal/below 40%, or greater than 40% is plotted. The expression of 8 target genes in class 1, 16 genes in class 4 and 1 gene in class 6 cannot be determined due to low target gene expression level or absence of sufficient germ cells. Thus the efficiency of these knockdowns cannot be evaluated accurately. The residual expression of 77 genes in class 1, 5 genes in class 2, 9 genes in 3, 3 genes in class 4 and 11 genes in class 6 was greater 40%. The residual expression of 23 genes in class 1, 4 genes in class 2, 13 genes in 3, 2 genes in class 4, 1 gene in class 5 and 16 genes in class 6 were equal or less than 40%. The residual expression of the target genes in the oocytes produced by RNAi females was examined using RT-PCR and band intensity quantification. Band intensity of the target amplicon was normalized to that of *Rpl32* in control and RNAi samples. The expression of genes cannot be evaluated when their knockdown abolished egg production, due to the lack of oocytes. The expression of 8 genes could not be detected with RT-PCR.

In our primary screen, all 207 TRiP lines, were screened using the MTD-Gal4 driver which drives shRNA production throughout oogenesis (Petrella *et al.* 2007). For the genes whose knockdown led to severely reduced or abolished egg production, we performed a secondary screen using *mata4*-GAL4-VP16, which drives shRNA expression in mid and late oogenesis (Radford *et al.* 2012, Sugimura & Lilly 2006). We expected that for genes that have crucial functions in early oogenesis, the temporal manipulation of RNAi knockdown could allow the depletion of gene products in mature oocytes without severely impacting egg production. We grouped the 189 genes into six classes according to their knockdown phenotypes in the primary and the secondary screen (Table 1, Figure 1A, Table S1).

**Table 1:** CLASSIFICATION OF FERTILITY PHENOTYPES IN THE PRIMARY AND SECONDARY GERMLINE SPECIFIC RNAi SCREEN

Class	MTD-Gal4*		*mata4*-GAL4-VP16**		Number of genes	Interpretation
	Egg production	Hatchability	Egg production	Hatchability		
**Class 1**	Normal	Normal	NA	NA	108	RNAi efficiency; not essential
**Class 2**	Reduced	Normal	NA	NA	9	Oogenesis
**Class 3**	Severely reduced	NA	Normal	Reduced	21	Early oogenesis; egg activation/ embryogenesis
**Class 4**	Severely reduced	NA	Severely reduced	NA	22	Mid to late/ throughout oogenesis
**Class 5**	Reduced	Reduced	Reduced	Reduced	1	Mid to late/ Throughout oogenesis; egg activation/ embryogenesis
**Class 6**	Normal	Reduced	NA	NA	28	Egg activation/ embryogenesis

* Germline-specific driver that drives sh-RNA production throughout oogenesis.

** Germline-specific driver that drives sh-RNA production in mid to late oogenesis.

The 81 genes in Classes 2 to 6 whose knockdown caused a range of defects in the female germline or early embryos will be discussed in more detail below. For the remaining 108 genes (Class 1), knockdown of their expression throughout oogenesis (MTD-Gal4 > UAS-shRNA) did not impact female fertility. RT-PCR for ∼71% of these 108 genes (*e.g.*, 77 genes) indicated low RNAi efficiency (residual expression > 40%; Figure 1B), suggesting that the currently available TRiP lines did not permit full evaluation of those genes’ roles in oogenesis or early embryogenesis. Ovary expression of eight genes was too low to be detectable under our RT-PCR conditions, making it difficult to assess their knockdown efficiency. For the 23 genes in Class 1 that knocked down to 40% or less of residual expression, the lack of phenotype might mean that they are not uniquely essential for female fertility, but we cannot rule out that the residual gene product was sufficient for their function.

#### Genes whose knockdown affect oogenesis:

For the nine genes in Class 2, depletion of their products led to slight reductions in egg production, but did not significantly influence the hatchability of the eggs. These genes may be involved in oogenesis, but their maternally-derived products are not essential thereafter. It is also possible that their expression was not sufficiently knocked down to fully block oogenesis (or to show later effects).

Genes in Class 3 and Class 4 are likely crucial for oogenesis since knockdown of their expression throughout oogenesis led to severe reduction or complete abolition of egg production. We examined the ovaries of the females knocked down for these genes and found defects at various stages of oogenesis (Figure S1, Table S3), validating that perturbing the expression of these genes in the female germline indeed disrupts various aspects of oogenesis. A summary of the ovary phenotypes can be found in Table S3. For the 21 genes in Class 3, restricting depletion of their gene products to mid and late oogenesis by driving knockdown with the *mata4*-GAL4-VP16 driver circumvented the impacts of their knockdowns on egg production and revealed various hatchability phenotypes. This subset of genes is thus likely important in early oogenesis, as well as in egg activation or embryogenesis, but their expression may not be essential in mid and late oogenesis. However, we cannot rule out the possibility that perdurance of protein products from early stages of oogenesis masked any defects from knockdown in later stages of oogenesis, or that knockdown of these genes driven by *matα4-*GAL4-VP16 was insufficient to generate an oogenesis phenotype.

For the 22 genes in Class 4, temporal manipulation of their knockdown did not alter the impact of these knockdowns on egg production. Thus, expression of these genes appears to be necessary throughout oogenesis for normal egg production, and it is impossible to determine whether they also affect egg activation or early embryogenesis. Knockdown of *Acn* significantly impacted both egg production and hatchability when driven by either driver, indicating its involvement in late oogenesis as well as egg activation or embryogenesis. Hence, we assigned *Acn* to a different class, Class 5.

#### Genes whose knockdown affect hatchability but not oogenesis:

For the 28 genes in Class 6, knocking down their expression throughout oogenesis significantly reduced or abolished the hatchability of the fertilized eggs without impacting egg production, suggesting that these genes’ activity is essential in egg activation and/or embryogenesis but not for oogenesis (Table 1, Figure 1A, Table S1).

To rule out effects of potential off-targets on our results, we obtained all available shRNA lines targeting a given gene. Of the 16 sets of shRNA lines that targeted the same genes, 11 generated the same phenotype in all lines in a set (Table S1). For the other 5 sets of shRNA lines, fertility phenotypes were observed in some lines but not the others in a given set. For four of those five sets, the difference in phenotypes was most likely due to differences in knockdown efficiencies, since the lines with no fertility defects showed the highest levels of residual expression. The two shRNA lines targeting *Nap1* generated similar levels of knockdown, thus the difference in phenotypes may be a result of small differences in knockdown that were undetectable by our semi-quantitative methods, to off-target effects of one of the constructs, or to unknown genetic or environmental factors that varied between the lines and their tests.

Taken together, the combination of MTD-GAL4 and *matα4-*GAL4-VP16 driven germline-specific RNAi identified 53 genes with apparent roles in oogenesis. This group includes factors known to mediate essential processes in oogenesis, such as *14-3-3ε (**Benton et al. 2002**)*, *no child left behind* (*nclb*) (Casper *et al.* 2011), *CG9556* (Pan *et al.* 2014) and *short stop* (*shot*) (Röper and Brown 2004), which validated the effectiveness of our method at capturing genes needed for egg production. Intriguingly, 32 of the 53 genes we found had no previously reported roles in oogenesis. These genes likely represent new factors for oogenesis that merit further investigation.

We also found 51 genes that are likely to be involved in egg activation and/or embryogenesis. The localization and/or cellular functions of the products of several of these 51 genes, including *Su(var)*205 (Zhao & Eissenberg 1999, Zhao *et al.* 2001), Modulo (Perrin *et al.* 1999), Otefin (Ashery-Padan *et al.* 1997, Habermann *et al.* 2012) and Axin (Tacchelly-Benites *et al.* 2018, Willert *et al.* 1999, Yamamoto *et al.* 1999), are known to be regulated through phosphorylation. Notably, 22 of these genes have no previously characterized maternal phenotypes.

### Maternal knockdowns of 27 genes caused developmental arrest in early embryogenesis

To investigate the roles of the maternal proteins whose knockdowns caused hatchability phenotypes (Classes 3, 5, and 6), we examined the phenotypes of 2-4-hr old embryos produced by RNAi females fertilized by wildtype (ORP2) males. Genes whose knockdown reduced the hatchability of the embryos to 50% or below were prioritized (43 genes).

**Figure 2:**
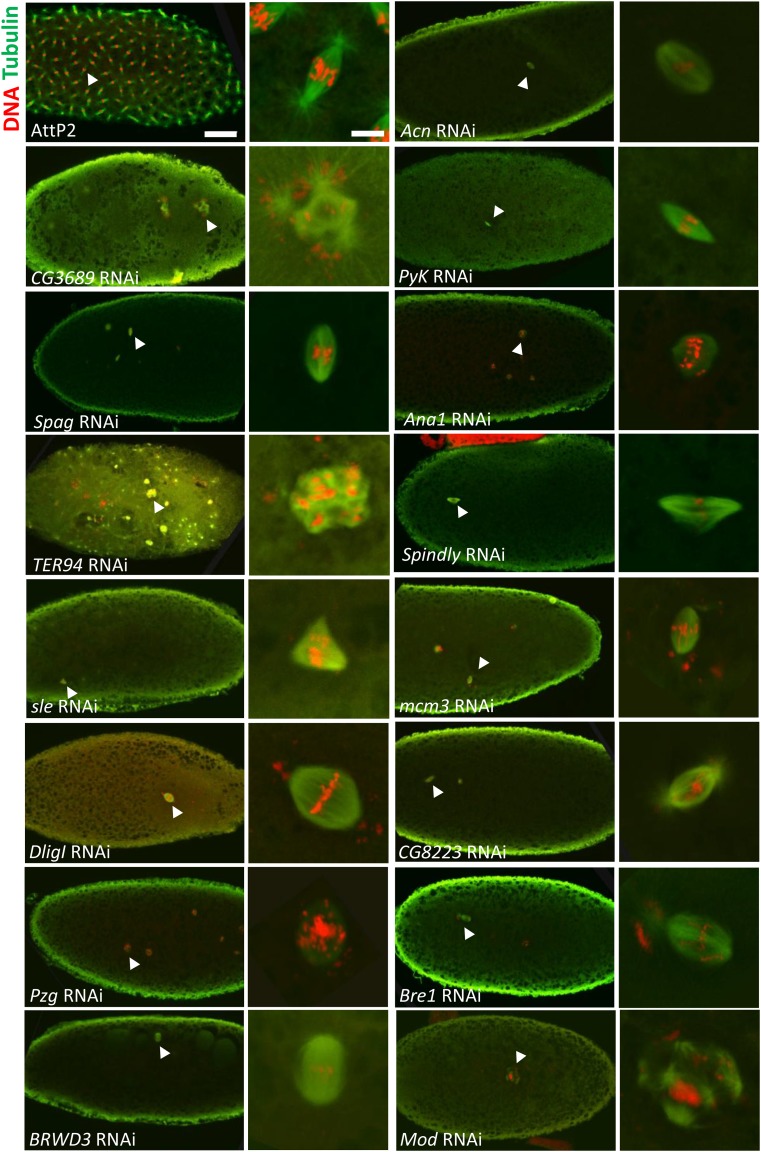
Maternal knockdown of 15 genes led to developmental arrest in stage 1 and/or early stage 2 of embryogenesis. 2-4 hr old embryos from knockdown females, stained for tubulin (green) and DNA (red). For each set of images, the one on the right shows a close-up view of the spindle (marked by the arrow on the left). Embryos from AttP2 control females mated with ORP2 males are used as positive controls. Scale bars: embryo 50 µm, nucleus 5 µm.

*D. melanogaster* embryos go through 14 syncytial mitotic divisions (embryogenesis stage 1-4) in the first 2 hr of development, before cellularization takes place at stage 5 of embryogenesis. Embryonic development before cellularization is largely maternally driven (Campos-Ortega and Hartenstein 1997). At 2-4 hr post fertilization, more than 80% of fertilized embryos produced by control females developed to stage 4 or later stages of embryogenesis (Table S4), whereas in the knockdown embryos, we observed developmental arrest at various earlier stages of embryogenesis (Figure 2, Figure S2, Table S4). We were especially interested in knockdowns that led to arrest at stage 1 (apposition of male and female pronuclei) or 2 (syncytial mitotic division 1-8) of embryogenesis, since early arrest likely reflects defects in egg activation or in the transition into zygotic syncytial divisions.

We observed that for 27 genes, maternal knockdown caused a significant portion of the embryos to arrest in stage 1 and/or early stage 2 of embryogenesis. Knockdown of 17 of these genes caused arrest in stage 1 of embryogenesis, prior to completion of first mitosis: *DNA ligase I* (*Dlig-I*) (83% arrested, n = 36), *minichromosome maintenance* (*mcm3*) (80% arrested, n = 25), *spindly* (54% arrested, n = 26), *spc105r* (91% arrested, n = 34), *ballchen* (*ball*) (89% arrested, n = 28), *BRWD3* (38% arrested, n = 60), *mbs* (40% arrested, n = 30), *mri* (87% arrested, n = 31), *pyruvate kinase* (*pyk*) (46% arrested, n = 48), *bre1* (43% arrested, n = 35), *mod(mdg4)* (58% arrested, n = 26), *slender lobes* (*sle*) (30% arrested, n = 27), *aubergine* (*aub*) (68% arrested, n = 28), *αTub67C* (96% arrested, n = 25), *acn* (36% arrested, n = 28), *spaghetti (spag)* (13% arrested, n = 72) *and modulo* (*mod*) (24% arrested, n = 55) (Table S4), indicating roles of these genes in egg activation or in initiation of syncytial division.

Maternal knockdown of 17 of the 27 genes caused significant embryogenesis arrest in early stage 2: *TER94* (86% arrested, n = 21), *spindly* (38% arrested, n = 26), *14-3-3ε* (86% arrested, n = 21), *pzg* (91% arrested, n = 22), *mbs* (40% arrested, n = 30), *su(var)205* (40% arrested, n = 15), *PyK* (52% arrested, n = 48), *mod(mdg4)* (42% arrested, n = 26), *aub* (32% arrested, n = 28), *sle* (67% arrested, n = 27), *MCPH1* (87% arrested, n = 23), *ana1* (100% arrested, n = 20), *pk92B* (42% arrested, n = 33), *plutonium* (*plu*) (71% arrested, n = 21), *spag* (19% arrested, n = 72), *CG8223* (61% arrested, n = 33), *CG3689* (40% arrested, n = 48). Note that the knockdown of seven genes: *mbs*, *sle*, *mod(mdg4)*, *spag*, *spindly*, *PyK* and *aub*, led to significant arrest in both stage 1 and stage 2 of embryogenesis; thus, they are included in both categories here. In addition, the knockdown of *eco* caused significant developmental arrest in late stage 2 (63% embryos) of embryogenesis.

We confirmed that the lack of hatchability was not due to failure of fertilization, by testing for the presence of a sperm tail in 0.5-1.5-hr old embryos produced by knockdown females (Karr 1991). We found no outstanding reduction in fertilization rate in any of the knockdown embryos (Figure S3, Table S5). Examination of the sequences of these 27 maternal effect genes revealed that many encode proteins with consensus phosphorylation sites for conserved kinases that are involved in or modulated during *Drosophila* egg activation, including Cdk1 (deactivated upon egg activation (Swan & Schupbach 2007)), Erk (activity decreases upon egg activation (Sackton *et al.* 2007)) and GSK3 (activity required for egg activation in Drosophila (Takeo *et al.* 2012)) (Table S6). The presence of these consensus sites suggests that these proteins may be targets of these kinases, consistent with their phospho-modulation during this transition, and suggesting the functional importance of their phosphoregulation at this time. Several of the proteins also contain consensus sites for CaMKIIγ, the calcium dependent kinase that plays essential roles in mammalian egg activation (Backs *et al.* 2010), but has not yet been tested for involvement in Drosophila egg activation.

Twelve of the 27 genes have previously reported maternal effect phenotypes, validating the premise of our screen that the phosphoregulated proteins include ones whose action is essential during this transition. These 12 genes include *ball* (Ivanovska *et al.* 2005, Nikalayevich & Ohkura 2015), *plu* (Elfring *et al.* 1997, Shamanski & Orr-Weaver 1991), *mod(mdg4)* (Buchner *et al.* 2000), *spc105r* (Radford *et al.* 2015, Yan *et al.* 2014), *mri* (Krauchunas *et al.* 2012), *su(var)205* (Kellum & Alberts 1995), *pk92B* (Sopko *et al.* 2014), *αtub67C* (Komma & Endow 1997, Matthews *et al.* 1993), *MCPH1* (Brunk *et al.* 2007), *mbs* (Sopko *et al.* 2014), *aub* (Mani *et al.* 2014), and *14-3-3ε* (Perrimon *et al.* 1996). In most cases, the knockdown phenotypes we observed recapitulated the previously reported maternal effect phenotypes; the few small discrepancies are likely due to incomplete RNAi efficiency. As an example, *ball* encodes a kinase that is crucial for the condensation of chromosomes during meiosis (Nikalayevich & Ohkura 2015). We observed that Ball depletion in the female germline caused 89% of her embryos to arrest before completing their first mitosis, with aberrant chromosome arrangements in the polar body. This resembles the previously reported phenotype observed in embryos produced by *ball* mutant females (Ivanovska *et al.* 2005, Nikalayevich & Ohkura 2015). Our recapitulation of phenotypes reported in previous studies using germline clones, RNAi, or hypomorphs validated the effectiveness of our methods in detecting genes with maternal effect phenotypes. A full summary of the phenotypes observed in maternally depleted embryos for the 27 genes can be found in Table S4.

Intriguingly, for 15 of the 27 genes no maternal effect phenotype had previously been reported in Drosophila. We discuss these genes in more detail below. Thirteen of these genes had previously been shown to affect cellular processes later in development; our results show that they are also critical in the germline for the initiation of embryogenesis. The phosphorylation state change of the maternal product of these genes upon egg activation reflects that they may be actively manipulated to adapt changes of roles during the transition from oocyte to embryo. This group also include two novel factors, CG3689 and CG8223, whose functions are not yet known in Drosophila. Our results revealed their essential involvement in early embryogenesis as maternal effect factors.

### Depletion of newly identified maternal effect factors causes spindle defects in early embryos

To further evaluate the functions of the maternal products of the 15 genes in early embryos, we examined the defects in 2-4 hr old embryos produced by females knocked down for each of these 15 genes (Figure 2, Table S4).

**Figure 3:**
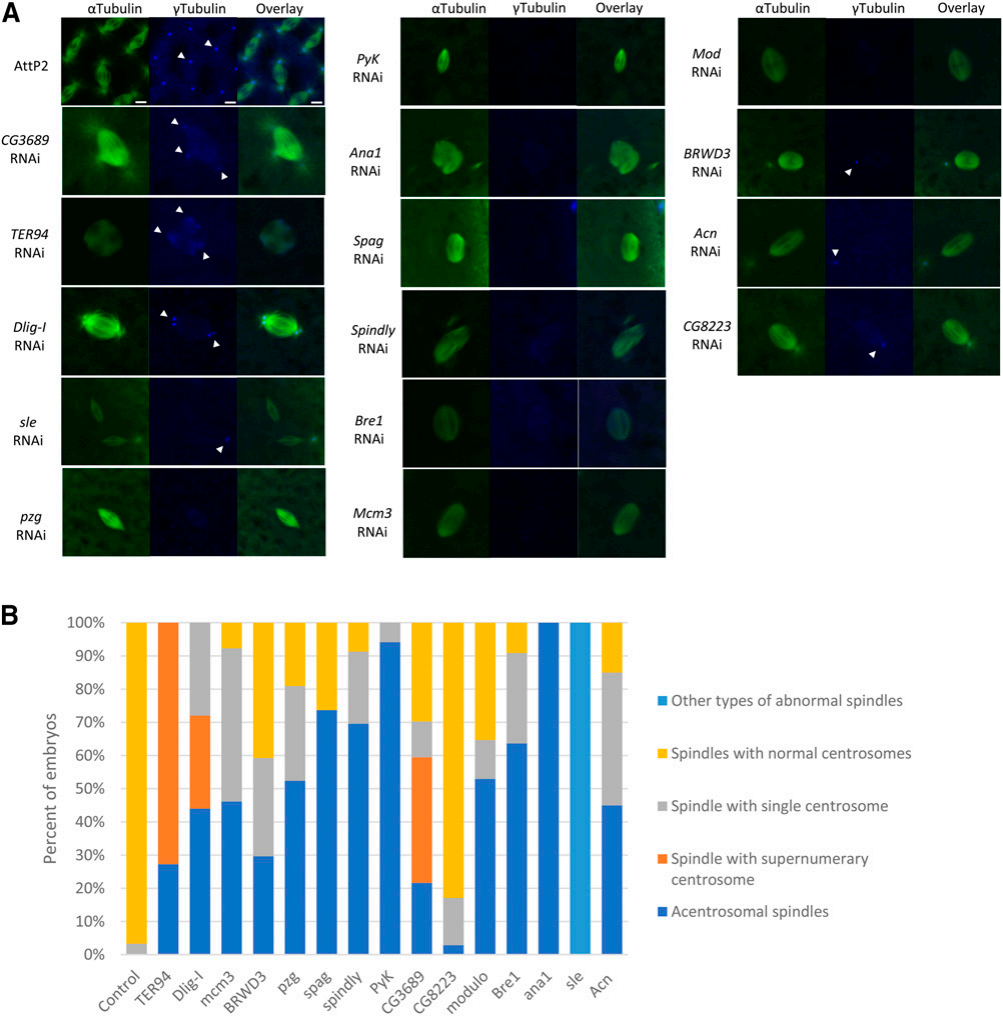
Maternal knockdown of 15 new maternal effect factors led to centrosome abnormalities. (A) Representative images of metaphase spindles in early embryos with normal (AttP2) or abnormal (maternal RNAi knockdowns) centrosome arrangement. 2-4hr old embryos are stained for αtubulin (green), and γtubulin (blue). Scale bar: 5µm. Gamma tubulin signals are marked by white arrows. (B) Proportion of maternal knockdown or control embryos with normal, supernumerary, acentrosomal spindles or spindle with single centrosome. A summary of the centrosome activity in the embryos with knockdown of these 15 genes can be found in Table S7.

Strikingly, for all 15 genes, depletion of their products from the female germline resulted in metaphase spindle structure abnormalities in early embryos, including multi-polar and anastral spindles (Table S7, Figure 3A). Since both types of defect are associated with abnormal centrosome activity, we examined the presence of centrosomes in the knockdown embryos by staining for the centrosome component γtubulin (Moritz *et al.* 1995).

Intriguingly, multi-polar spindles with supernumerary centrosomes were observed in 73% of *TER94* and 38% of *CG3689* maternal knockdown embryos (Figure 3B), suggesting that over-replication of centrosomes may be associated with aberrant spindle formation in these embryos. Consistent with this observation, we also found excessive accumulation of centrosomes unassociated with spindles in the cytoplasm of these embryos, suggesting uncoupling of centrosome replication from the cell cycle.

TER94 is a VCP family protein that is involved in microtubule organization and mRNA localization during Drosophila oogenesis (Ruden *et al.* 2000). However, its roles in early embryogenesis are unclear. CG3689 is the fly ortholog of mammalian NUDT21, which is an RNA processing factor that regulates differential mRNA polyadenylation in differentiating stem cells (Brumbaugh *et al.* 2018, Dettwiler *et al.* 2004, Rüegsegger *et al.* 1998, Yang *et al.* 2010). The function of CG3689 in Drosophila is yet unknown beyond our finding that it is required maternally for mitotic spindle organization in the early embryo.

γtubulin-positive centrosomes also accumulated in the embryos produced by *Sle* RNAi females. However, instead of spindles with supernumerary centrosomes, we found a mixture of normal spindles and spindles with no or one single centrosome in all *Sle* knockdown embryos (Figure 2, Figure 3A), suggesting that even though centrosomes over-replicated in these embryos, they were not able to properly interact with chromosomes to form mitotic spindles. Sle is known to be involved in nucleolar organization and rRNA processing in neuron and nurse cells (Orihara-Ono *et al.* 2005). Our results reveal a novel function for Sle, as a maternal protein that is crucial for the mitotic divisions in early embryogenesis.

Spindles with single centrosomes were also observed in embryos from females knocked down for *Mcm3* (46%), *Acn* (40%), *BRWD3* (30%), *pzg* (29%), *Dlig-I* (28%), *Bre1* (27%), *spindly* (22%), *CG8223* (14%), *mod* (12%) and *CG3689* (11%) (Figure 3B), suggesting abnormal centrosome-chromosome interaction in these knockdown embryos.

Another commonly observed spindle defect in the early embryos from maternal knockdown of these 15 novel maternal effect genes is an acentrosomal spindle. We found bipolar spindles with no centrosomes at spindle poles in large portions of embryos with maternal knockdown of *Ana1* (100%), *PyK* (94%), *Spag* (74%), *Spindly* (70%), *Bre1* (64%), *Mod* (53%), *pzg* (52%), *Mcm3* (46%), *Acn* (45%), *Dlig-I* (44%), and *BRWD3* (30%) (Figure 3B). The absence of centrosomes at spindle poles may be a result of unsuccessful centrosome replication or aberrant interaction between centrosomes and chromosomes. Among this group of genes, *Ana1* encodes a centrosome component known to be present in the sperm’s giant centriole and proximal centriole-like structure (Blachon *et al.* 2009). Our result suggests that maternal contribution of Ana1 is crucial for the replication of centrosomes in early embryos. Other genes in this group are not known to be involved in centrosome activities or spindle formation.

### Maternal depletion of PyK affected meiosis completion

Since acentrosomal spindles are characteristic of meiotic spindles, another possibility for the presence of abnormal spindles in these embryos is that meiosis is not completed. We thus examined the status of meiosis completion in the knockdown embryos.

In *Drosophila melanogaster*, completion of meiosis in activated eggs is accompanied by the fusion of three polar bodies into a rosette structure, with condensed chromosomes surrounded by microtubules (Mahowald *et al.* 1983). Therefore, we searched for the presence of a polar body rosette in 0.5-1.5hr old embryos as a signature of meiosis completion (Table S5).

**Figure 4:**
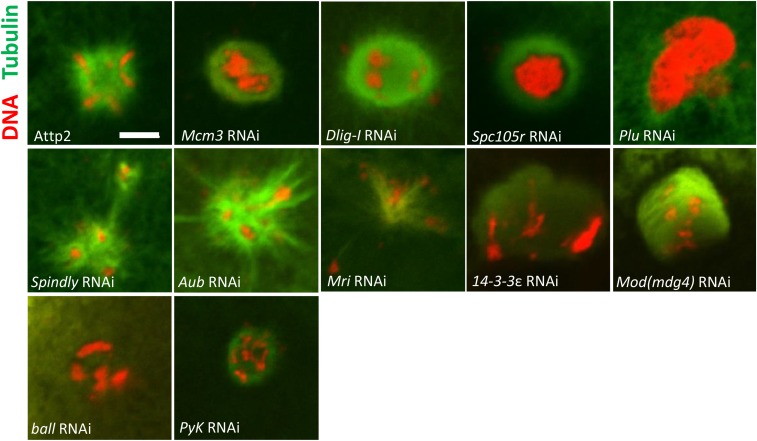
Polar bodies with abnormal morphology were observed in knockdown embryos for 11 genes. Significant portions of 0.5-1 hr embryos with maternal knockdown of *Dlig-I* (83% abnormal, n = 23), *mcm3* (56% abnormal, n = 36), *spc105r* (96%, n = 23), *plu* (100%, n = 30), *spindly* (53% abnormal, n = 32), *aub* (57%, n = 28), *mri* (62% abnormal, n = 37), *14-3-3ε* (93% abnormal, n = 29), *mod(mdg4)* (58% abnormal, n = 24), *ball* (88%, n = 24) and *PyK* (13%, n = 31) had polar bodies with abnormal morphology. Tubulin is shown in green. DNA is shown in red. Scale bars: 5µm.

Normal polar body rosettes were observed in 93% of the embryos produced by control (AttP2) females (Figure 4). For 9 of the 15 genes, we found no major aberrations in the presence or morphology of polar body rosettes in the embryos produced by RNAi females (Table S5). But strikingly, polar body rosettes were absent in large proportions of embryos from females with germline knockdowns of *PyK* (65% absent, n = 31) consistent with the scenario of incomplete meiosis. *PyK* encodes pyruvate kinase, an important component of the glycolytic glycolysis pathway. Our results suggested that glycolysis, and its regulation, may be essential for egg activation.

We also found prominent abnormalities in polar body morphology in embryos with maternal knockdown of *Dlig-I* (83% abnormal, n = 23), *mcm3* (56% abnormal, n = 36), and *spindly* (53% abnormal, n = 32) (Figure 4), indicating possible defects in chromosome condensation and microtubule organization during the formation of polar body rosettes in these knockdown embryos.

A complete summary of polar body rosette presence can be found in Supplementary Table S5.

### Maternal knockdown of Acn leads to defects in the crosslinking of egg coverings

Early embryogenesis arrest may reflect defects in egg activation or in the transition into zygotic syncytial divisions (or both). Thus, we investigated whether the maternal knockdowns that caused early developmental arrest had any impacts on egg activation events, in addition to meiosis completion. Specifically, we examined two other major events of egg activation in the embryos produced by the knockdown females: changes in egg coverings and translation initiation.

In *Drosophila melanogaster*, egg activation is accompanied by the cross-linking of the vitelline membrane outer layer and the chorion, causing the egg coverings to become impermeable to small molecules (Heifetz *et al.* 2001), and renders the eggs resistant to rapid lysis (in <2 min) in 50% bleach. Thus, to examine whether hardening of the egg coverings was affected by any of the 27 early arrest genes, we examined the lysis of 0.5-1.5-hour-old embryos from knockdown (and control) females after incubation in 50% bleach. We found that the maternal knockdown of *Acn* caused a significant reduction in bleach resistance; only 41% of eggs produced by *Acn* knockdown females persisted longer than 2 min in 50% bleach (Table S5), suggesting a role for Acn in facilitating eggshell production or mediating its cross-linking. Maternal knockdown of the other 26 genes did not significantly affect the bleach resistance of the eggs. *Acn* is important for endosomal trafficking and for the stabilization of early endosomes (Haberman *et al.* 2010). It is also involved in RNA splicing as an accessory factor of the exon junction complex (Hayashi *et al.* 2014).

Egg activation is also accompanied by a dramatic increase in the translation of many maternal mRNAs important for subsequent embryogenesis (Horner 2007, Kronja *et al.* 2014b), including (in Drosophila) *smaug* (*smg*), *bicoid* (*bcd*) and *zelda* (*zld*) (Driever & Nusslein-Volhard 1988, Eichhorn *et al.* 2016, Tadros *et al.* 2007). We thus examined the translation of *smg* in activated eggs as a marker of a general increase in translational activities. Since Drosophila egg activation is independent of fertilization (Horner & Wolfner 2008a), we used 0-1hr activated but unfertilized eggs produced by knockdown females for this experiment to avoid the confounding influences of fertilization and subsequent zygotic development. As expected, Smg protein was detected in the lysate of activated eggs but not in lysates of stage 14 oocytes produced by control females (Tadros *et al.* 2007). We did not detect Smg protein in activated eggs produced by females knocked down for *plu*. This was expected, because *plu* encodes a subunit of the Pan Gu Kinase that is essential for translation of Smg upon egg activation (Tadros *et al.* 2007). In contrast, we found no significant disturbance of *smg* translation in eggs knocked down for any of the other 26 maternal knockdowns, indicating that none of those genes are essential for the translational increase that normally occurs upon egg activation (or for the translation of Smg specifically) (Figure S4). We cannot rule out the possibility that the 26 genes whose knockdown did not affect *smg* translation may include factors that regulate the translation of specific maternal mRNAs other than *smg*, or that their knockdown level was insufficient to give a detectable effect on *smg* translation.

## Discussion

In recent decades, germline-specific RNAi screening of genes with enriched germline expression has been used in several studies to identify genes that are important for female fertility. An RNAi screen of this type in *C. elegans* revealed that 322 out of 766 genes with ovarian enriched expression are required for normal egg production and/or embryogenesis (Piano *et al.* 2002). A similar screen in Drosophila found that 10.5% of the 3491 genes with germline-enriched expression were important for female fertility (Yan *et al.* 2014). However, since not all genes that are important for female fertility are preferentially expressed in the female germline, limiting screens to germline-enriched genes may miss important regulators of female fertility that are more uniformly expressed, such as genes that are required for cell-essential functions in the soma later in development. Our study addressed this limitation by searching for maternal functions among proteins that are phosphoregulated during egg activation. Of 189 candidate genes, we identified 81 that are crucial for different aspects of female fertility, indicating that this set of phosphoregulated maternal proteins is a rich candidate pool of important modulators of oogenesis and early development.

Our screen identified 27 genes that encode essential maternal proteins for the egg-to-embryo transition or early in embryogenesis. This set of genes includes several that are essential for cellular processes including mitosis, DNA damage repair and replication in later somatic cells, but for many this is the first report that they also exert these functions maternally, or in the germline. Since early embryogenesis in Drosophila takes place with little transcription activity, it is possible that the products of these essential genes need to be provided maternally to facilitate cellular functions at these early stages.

In this screen, we did not find new germline-specific regulators of female fertility, but rather we revealed germline functions for potentially ubiquitous proteins. Although genetic screens have identified some female-germline-specifically expressed molecules that are essential for the egg-to-embryo transition, such as *fs(1)YA* (Lin & Wolfner 1991, Liu *et al.* 1995), *gnu* (Freeman & Glover 1987, Renault *et al.* 2003), *plu* (Elfring *et al.* 1997, Shamanski & Orr-Weaver 1991), *png* (Hara *et al.* 2017, Shamanski & Orr-Weaver 1991), and (almost exclusively) *wisp* (Benoit *et al.* 2008, Brent *et al.* 2000, Cui *et al.* 2008, Lim *et al.* 2016), our results indicate that this type of gene is rare and that most of the critical molecules for this transition are expressed, and often essential, at other stages. Detection of those molecules among proteins whose phosphorylation state changes during egg activation suggests that the activity of these essential maternally provided factors may be regulated post-translationally during the transition from oocyte to embryo to allow them only to be active at the appropriate time. This will be a fruitful area for future molecular study.

In addition to discovering new, maternal, roles for known proteins, we found two novel factors, *CG3689* and *CG8223*, to be important for early syncytial divisions. Both proteins are conserved in mammalian systems. The mammalian ortholog or *CG3689* is *Nudt21*, which plays a role in the 3′ polyadenylation of pre-mRNAs (Dettwiler *et al.* 2004, Rüegsegger *et al.* 1998, Yang *et al.* 2010). CG3689 shares 77% amino acid sequence homology as NUDT21. Since maternal transcripts are dynamically controlled through poly(A)-tail length in late oogenesis and early embryogenesis (Benoit *et al.* 2008, Brent *et al.* 2000, Cui *et al.* 2008, Lim *et al.* 2016), it is possible that CG3689 plays a similar role to Nudt21 We frequently observed misshapen multi-polar spindles in *CG3689* knockdown embryos. Interestingly, embryos from females lacking *Wispy*, a Drosophila poly-(A) polymerase that is known to mediate polyadenylation of maternal transcripts upon egg activation, also arrest in early embryogenesis with abnormal spindle formation (Brent *et al.* 2000, Cui *et al.* 2008). If CG3689 plays a similar molecular role in Drosophila to NUDT21 in mammals, perhaps maternal transcripts of some spindle-associated proteins are regulated by CG3689, and the phenotype that we observe in its knockdown is due to the perturbation in production of some of these spindle-associated proteins. Though we did not detect abnormalities in Smg translation in CG3689 maternal knockdown, we cannot rule out that its function affects transcripts other than *smg*. This possibility is supported by the finding that Nudt21 binds to RNA at specific sequences (Yang *et al.* 2010). Examination of the global changes in maternal mRNA poly-A tail length upon egg activation in CG3689 depleted background will be informative to elucidate the functions of CG3689.

The mammalian ortholog of CG8223 is NASP (29% amino acid sequence identity), which is, surprisingly, a sperm-specific protein. NASP is a N1/N2 family histone chaperone involved in nucleosome assembly after DNA replication is completed (Finn *et al.* 2008, Osakabe *et al.* 2010, Richardson *et al.* 2006). This suggests interesting possibilities for the role of *CG8223* in Drosophila, as the phenotype we observed in *CG8223* knockdown embryos is consistent with disruptions in chromosome organization or chromatin assembly. Intriguingly, our finding suggests that this novel protein may be particularly important in early embryogenesis, as its depletion in early oogenesis did not seem to disrupt the formation of 16-cell germline cysts.

We were also intrigued to discover 13 genes that are required in early oogenesis and are also important for egg activation or early embryogenesis. Five of these genes encode factors involved in chromatin organization, including *su(var)205* (Hines *et al.* 2009), *pzg* (Eggert *et al.* 2004, Gan *et al.* 2011), *BRWD3* (Chen *et al.* 2015), *ball* (Aihara *et al.* 2004, Ivanovska *et al.* 2005) and *bre1* (Bray *et al.* 2005, Xuan *et al.* 2013). The activity of these factors may need to be regulated appropriately to allow the dynamic reorganization of chromatin landscape that accompanies the differentiation of the oocyte during oogenesis (Boija & Mannervik 2015, Iovino 2014), followed by conversion to totipotency during egg activation. Understanding how phosphoregulation modulates the activities of these factors and the kinases and other pathways involved in the phosphoregulation of these factors in response to the calcium signal that initiates egg activation will give valuable information about the mechanisms that facilitate the transition between differentiated and totipotent cellular states.

We also found evidence that the function and regulation of glycolysis are essential for egg activation since PyK appears to be dephosphorylated during egg activation (Krauchunas *et al.* 2012), and its depletion led to defects in meiosis completion. A recent study reports a drastic shift of metabolic state in late oogenesis, which is later reversed in the early embryo, pointing out that remodeling of metabolic state may be an important part of the transition from oocyte to embryo (Sieber *et al.* 2016). Since PyK is a component of the glycolysis pathway, its phosphoregulation may be a part of the mechanism that mediates the metabolic changes that take place during egg activation. Recently, it has also been reported in mouse that transient nuclear localization of TCA cycle enzymes in the zygote is essential for epigenetic remodeling of the zygotic genome upon zygotic genome activation (Nagaraj *et al*. 2017), suggesting another potential role for Pyk1 in the egg-to-early-embryo transition.

Guo *et al.* (2015) observed 174 proteins in common between our dataset of proteins phospho-modulated during Drosophila egg activation (Krauchunas *et al.* 2012) and their set of phosphoregulated proteins during this same transition in sea urchin. This led them to suggest that there are conserved cassettes of protein functions needed for this important developmental transition. With this in mind, we find it intriguing that 88% of the genes that our RNAi screen identified as needed for the transition from egg to early embryo in Drosophila have homologs in mouse. Interestingly, the mouse orthologs of seven of these genes (*ball* (mouse *vrk1*) (Wiebe *et al.* 2010), *MCPH1* (mouse *mcph1*) (Liang *et al.* 2010), *14-3-3ε* (mouse *ywhae*) (Kosaka *et al.* 2012), *rec* (mouse *mcm8*) (Lutzmann *et al.* 2012), *RhoGap68F* (mouse *Arhgap1*) (Wang *et al.* 2005), *spindly* (mouse *spdl1*) (Zhang *et al.* 2010) and *CG9556* (mouse *cops2*) (Lykke-Andersen *et al.* 2003)) have been reported to be important for female fertility, consistent with the idea of conserved functions in female reproduction. For the vast majority of the Drosophila fertility factors uncovered by our screens, we do not yet know the roles of their orthologs in female fertility in other organisms, and they are good candidates for investigation as regulators of egg activation or early embryogenesis in organisms beyond Drosophila.

For 108 of the genes that we tested, depletion of their products in the female germline did not impact female fertility. There are numerous explanations for this result, beyond the possibility that these genes might not be essential in these processes. First, in more than 71% of the cases, the knockdown efficiency of these RNAs was too low to allow definitive conclusions about gene function. Although in the future one could imagine circumventing this problem with screens using targeted editing with CRISPR/Cas9, at present those techniques are not sufficiently efficient to allow homozygous germline-specific knockouts. Second, even in the cases of high knockdown efficiency, lack of phenotype could reflect functional redundancy. Third, it is possible that some of these genes encode proteins that are not essential for oogenesis, but must be deactivated upon egg activation, or proteins that are needed to prevent premature activation of oocytes; neither type would have been detectable in our screen. Finally, the 311 proteins identified by Krauchunas *et al.* 2012 are unlikely to represent the entire set of proteins that are phosphoregulated upon egg activation since even the newer improved mass spectrometry currently available (5 years after that study) still cannot comprehensively detect all peptides in a sample. Therefore, additional essential factors that are phosphoregulated at egg activation likely remain to be discovered.
